# Beyond Lectures: Reimagining Psychiatric Didactics for the Age of AI

**DOI:** 10.2196/78110

**Published:** 2025-10-31

**Authors:** Laurent Elkrief, Alexandre Hudon, Giovanni Briganti, Paul Lespérance

**Affiliations:** 1 Département de Psychiatrie et d’Addictologie Faculté de Médecine Université de Montréal Montréal, QC Canada; 2 Centre Hospitalier de l’Université de Montréal Montreal, QC Canada; 3 Department of Psychiatry Institut universitaire en santé mentale de Montréal Montréal, QC Canada; 4 Department of Psychiatry Institut Philippe Pinel de Montréal Montreal, QC Canada; 5 Groupe Interdisciplinaire de Recherche sur la Cognition et le Raisonnement Professionnel Université de Montréal Montréal, QC Canada; 6 Service de Médecine computationnelle et neuropsychiatrie Faculté de Médecine, Pharmacie, et Sciences Biomédicales University of Mons Mons Belgium; 7 Département des Sciences Cliniques Faculté de Médecine University of Liège Liège Belgium

**Keywords:** large language models, medical education, didactic lecture, artificial intelligence, educational technology

## Abstract

The increasing use of generative large language models (LLMs) necessitates a fundamental reevaluation of traditional didactic lectures in medical education, particularly within psychiatry. The specialty’s inherent diagnostic ambiguity, biopsychosocial complexity, and reliance on nuanced interpersonal skills demand an educational model that transcends mere information transfer, focusing instead on cultivating sophisticated clinical reasoning. This viewpoint argues for a shift from passive knowledge transmission to active, facilitated development of higher-order thinking, aligning with the Bloom taxonomy. We describe four core propositions: (1) shifting foundational knowledge acquisition to faculty-curated asynchronous artificial intelligence (AI)–assisted micromodules; (2) transforming synchronous time into “Ambiguity Seminars” for discussing nuanced cases, biopsychosocial formulation, and ethical dilemmas, leveraging faculty expertise in guiding reasoning; (3) integrating live LLM critical interaction drills to develop prompt engineering skills and critical appraisal of AI outputs; and (4) realigning assessment methods (eg, objective structured clinical examinations [OSCEs], reflective writing) to evaluate clinical reasoning and integrative skills rather than rote recall. Successful implementation requires comprehensive faculty development, explicit institutional investment, and a phased approach that addresses scalability across varying resource settings. This reimagined approach aims to cultivate clinical wisdom, equipping psychiatric trainees with adaptive reasoning frameworks essential for excellence in an AI-mediated future.

## Introduction

The advent of powerful, publicly accessible large language models (LLMs) like ChatGPT marks an inflection point for medical education. Specifically, these tools are driving a shift from information scarcity to abundance, which directly challenges the traditional role of the didactic lecture as the main medium of information transfer. Consequently, the widespread adoption of these tools necessitates a fundamental rethinking of this lecture model. The necessity to evolve beyond traditional didactics is amplified in psychiatry, a specialty with inherent diagnostic ambiguity, profound biopsychosocial complexity, and a fundamental reliance on nuanced interpersonal competencies and the interpretation of subjective human experience. These defining features demand an educational model that transcends mere information, one that actively cultivates the sophisticated clinical reasoning and integrative skills essential for practice. Such a model aligns with established pedagogical frameworks like the Bloom taxonomy, aiming to engage trainees across a spectrum of cognitive processes, from foundational understanding to higher-order thinking and complex problem-solving. Consequently, we argue that psychiatric didactic time must pivot from passive knowledge transmission toward an active, facilitated development of clinical reasoning, with faculty evolving from information repositories (“sage on the stage”) [1] into catalysts for critical thinking and contextualization. This proposed evolution aligns with the core tenets of competency-based medical education, which prioritizes the demonstration of integrated professional capabilities over time-based training and simple knowledge acquisition [2]. It is a direct application of the call to cultivate “adaptive expertise,” the ability to flexibly and innovatively apply deep conceptual knowledge to novel problems, which stands in contrast to the routine expertise fostered by traditional didacticism [3]. This viewpoint outlines four core propositions for this necessary transformation.

Psychiatry’s inherent complexity imposes a high intrinsic cognitive load, yet traditional lectures often increase extraneous load through passive delivery, hindering deep processing and schema learning [4]. Seeking greater efficiency, trainees increasingly forgo these sessions. This mismatch contributes significantly to declining attendance as trainees prioritize the flexibility of alternative resources [5]. Beyond attendance effects, meta-analyses indicate that active learning consistently improves achievement and reduces failure compared with traditional lectures [6], with flipped designs in medical education showing measurable gains when preclass work is structured and accountability is built in [7].

## The Shifting Landscape: AI’s Impact on Medical Education and Psychiatry’s Unique Needs

The widespread availability of LLMs for educational purposes [8-10] drastically accelerates this trend by collapsing traditional knowledge asymmetries. However, alongside potential benefits, the unguided use of these tools necessitates critical artificial intelligence (AI) literacy due to inherent risks of these systems, mainly around inaccuracies and “hallucinations” [10-15] as well as the potential propagation of embedded societal biases [16-18]. Additionally, the risk of automation bias influencing clinical judgment [13], coupled with outputs often lacking the critical nuance essential for psychiatric practice [10,15,16], further underscores current AI limitations. Effectively navigating this landscape demands not only critical evaluation skills but also proficiency in prompt engineering [14,19]. Current didactic structures, largely reliant on outdated lecture formats, are ill-equipped for this complex new reality, failing to prepare trainees for an information environment increasingly mediated by AI. This challenge is not unique to the AI era; for decades, educational theorists have argued for the necessity of active learning methodologies to move beyond the passivity of the lecture format and better cultivate the complex reasoning skills required for professional practice [1]. This aligns with meta-analytic evidence from health profession education and the broader higher education literature showing flipped/active approaches outperform lecture-based formats [6,7].

Beyond general AI challenges, psychiatric education faces unique demands. Diagnosis relies on subjective interpretation and negotiated constructs, not definitive tests, with evolving models adding complexity. Effective practice requires sophisticated biopsychosocial formulation, integrating diverse data (biology, psychology, narrative, and social context), which is a reasoning skill poorly served by simple fact delivery. Current LLMs struggle with the nuance, empathy, subjectivity, and deep biopsychosocial integration vital for psychiatry [15,16,20]. Given that navigating uncertainty and ambiguity are core competencies, psychiatric education must prioritize cultivating robust clinical reasoning, metacognition, and critical thinking to develop “clinical wisdom” over mere recall [1,4].

Developing such clinical wisdom demands a pedagogical evolution. Because LLMs reduce the challenge of accessing factual knowledge, faculties’ comparative advantage shifts to fostering higher-order cognitive skills such as critical thinking, contextual reasoning, and the synthesis of information, evolving their role from primary knowledge sources to catalysts for these deeper learning processes. The following propositions operationalize this shift.

## Core Propositions for Reimagining Psychiatric Didactics

First, the acquisition of foundational knowledge, corresponding to initial cognitive levels in the Bloom taxonomy such as “Remembering and Understanding,” shifts to faculty-curated AI micromodules. These short asynchronous resources, perhaps AI-drafted [15,19,20] but rigorously vetted for accuracy/nuance [10,14-16], free synchronous time and reduce extraneous cognitive load [21,22]. This vetting process would involve cross-referencing AI-generated content against established clinical guidelines and seminal texts, scrutinizing for embedded biases [16-18], verifying the authenticity of citations [11], and ensuring the material aligns with local practice standards and the appropriate learner level.

Second, synchronous time becomes an “Ambiguity Seminar,” where psychiatric complexity is addressed directly. Faculty use nuanced vignettes to teach how to reason, framing the clinical problem and uncertainties first, rather than delivering more facts [1]. To achieve this, faculty would use techniques such as Socratic questioning to probe assumptions and guide hypothesis generation (“What evidence supports that diagnosis over others?”). They would also focus on metacognitive modeling, verbalizing their own reasoning process when faced with uncertainty (“Here is why I am prioritizing this intervention, despite these conflicting data points...”) to demonstrate how experts navigate ambiguity, thereby shifting the focus from finding a single correct answer to developing a robust and defensible reasoning process. While AI may help draft initial cases [18], instructors refine them toward situations in which diagnoses straddle categories and pharmacological guideline algorithms do not neatly apply. Learners then generate competing hypotheses with confirming and disconfirming data, craft a succinct biopsychosocial formulation, and propose a first-line plan that goes beyond the textbook, stating trade-offs and safety contingencies in the patient’s context. LLMs can be used as a sounding board, but outputs are treated as claims to be tested; their breakdowns become teachable moments about limits and bias. The aim is disciplined, creative problem-solving, yielding brief original formulations and plans that learners can defend aloud.

Third, seminars integrate live LLM critical interaction drills. Trainees query an LLM with case questions, then critique the output: checking accuracy, bias, relevance, and citations. This requires prompt engineering instruction [14,19,20]. Engaging in such exercises helps trainees develop sound habits for evaluating information, improves their AI literacy, and equips them to counter automation bias [13]. Prompting students to critique LLM responses encourages them to use the AI as a sounding board to refine their own clinical judgment; this process also lessens the risk of AI-driven inaccuracies and develops crucial practical abilities.

Finally, to ensure learning objectives are met, assessment must be realigned with reasoning skills, moving beyond recall. While the Bloom taxonomy can delineate how AI might assist with foundational knowledge tasks, evaluation must focus on higher-order thinking that is inherently resistant to artificial augmentation. Specifically, objective structured clinical examinations (OSCEs) should be designed to assess not just the analysis of complex information but also the trainee’s real-time interpersonal skills and their ability to adapt to unexpected information from a standardized patient. To ensure integrity, these OSCEs must be conducted in proctored environments where the use of external AI tools is prohibited. Similarly, reflective writing assignments can be made more robust by requiring trainees to integrate highly specific, personal patient interactions—details an AI could not fabricate—or by using in-class timed “reflection stems” that demand immediate synthesis of a shared experience. A mandatory oral defense of these reflections then becomes a nonnegotiable component to validate the authenticity of the reasoning and personal insights presented. These methods, by directly targeting the upper echelons of the Bloom taxonomy and evaluating skills requiring embodied clinical presence and personal experience, offer a more authentic assessment of the competencies that current AI systems struggle to replicate.

## A Framework for Implementation: Addressing Practical Challenges

We proposed a pragmatic blueprint that flips factual acquisition to curated micromodules, reclaims synchronous time for faculty-facilitated ambiguity seminars, and integrates AI-critical drills, with assessments aligned to higher-order clinical reasoning. This section aims to translate that design into an implementable institutional plan while acknowledging costs and constraints.

Translating these propositions from theory into practice requires a pragmatic strategy that directly addresses the significant challenges of institutional inertia, resource allocation, and faculty development. A successful rollout is not merely a technical task but a complex exercise in change management. The most significant barrier is often faculty resistance, which may stem from the substantial workload of curriculum redesign and a perceived evaluation of traditional lecturing expertise. Consequently, this change must be framed as an elevation of the faculty role, shifting members from information transmitters to expert guides who model and cultivate complex clinical reasoning. We acknowledge that this viewpoint presents a theoretical framework and that its efficacy has yet to be established through empirical research; its primary goal is to provide a road map for such investigation.

To manage this transition, a dedicated faculty development program is essential, requiring protected (and renumerated) time for hands-on training in advanced Socratic facilitation for the ambiguity seminars, critical AI literacy for appraising model outputs, and the skills for curriculum cocreation. Furthermore, this educational model is not resource-neutral and requires explicit institutional investment. Success is contingent on access to a stable and user-friendly learning management system; privacy-compliant AI platforms; and most critically, formally protected faculty time. This work cannot be an unfunded mandate added to existing clinical and academic responsibilities. To centralize and sustain the effort, programs might consider creating a dedicated role, such as a clinical AI education lead. Recognizing that institutional capacities vary widely, this framework is designed to be scalable. High-resource programs might implement the full model, while lower-resource settings can adopt a “low-fidelity” version using freely available language models and multi-institutional consortia for open-access materials. Crucially, this scalability must extend to individual trainees to ensure equitable participation. Programs should provide accessible, screen reader–friendly materials and use privacy-compliant AI platforms, offering device support or non-LLM analytic pathways where live access is infeasible. By embedding these accessibility measures, the core principles of flipping the classroom and focusing synchronous time on facilitated reasoning can be maintained inclusively across all settings.

Central to this framework is a commitment to educational equity. The integration of AI tools risks exacerbating existing disparities related to socioeconomic status, disability, or access to technology. Therefore, programs must actively ensure equitable implementation. This includes providing institutional access, where possible, to privacy-compliant AI platforms to avoid financial barriers for trainees; offering device support; and ensuring all digital materials are fully accessible and screen reader–friendly. Furthermore, the development of “non-LLM analytic pathways” is crucial; these are alternative assignments that achieve the same core learning objectives of critical reasoning and evidence appraisal but do not require live AI interaction, ensuring that technological barriers do not impede a trainee’s educational progress.

Finally, a phased 3-year timeline can make this significant reform manageable. Year one would focus on a pilot within a single teaching block to establish the proof of concept and gather feasibility data. Year two would involve expansion and refinement, using pilot data to roll the model out to other blocks. Year three would target full integration and sustainability, making the model standard practice and shifting research toward longitudinal multisite evaluation to assess broader generalizability.

Beyond implementation, systematic and rigorous evaluation is essential. Pilot studies should assess primary outcomes (OSCEs for formulation/reasoning/appraisal, reflective writing) and secondary measures, including engagement versus historical data [5], ambiguity tolerance, and satisfaction [1]. Process evaluation and qualitative focus groups should explore reasoning, AI trust, and cognitive load [4]. Future research needs longitudinal tracking, cost-utility analysis, AI comparisons, and scalability assessments, prioritizing methodological rigor [9,14] to address gaps in psychiatric AI education research [15,16].

## Conclusion

This reimagined approach aims to redefine psychiatric education for a new era defined by the widespread availability of knowledge through LLMs. Faculty should pivot from primarily dispensing facts toward cultivating clinical wisdom, defined as sound judgment under uncertainty. Accordingly, this viewpoint proposes retiring lectures focused on the transfer of facts in favor of curated micromodules, thereby reclaiming synchronous time for facilitated reasoning seminars that incorporate critical AI interaction. We hope programs pilot this model (or similar ones), focusing didactic time on core competencies like biopsychosocial formulation and ethical deliberation. Equipping trainees to interrogate machines, not just query them, requires moving beyond outdated methods. In an AI-mediated future, the cultivation of adaptive reasoning frameworks will be fundamental to clinical excellence.

## Appendix

Multimedia Appendix 1 Description of the use of generative artificial intelligence by the authors.

## Abbreviations

AI: artificial intelligence
LLM: large language model
OSCE: objective structured clinical examination
